# LncRNA PlncRNA-1 accelerates the progression of prostate cancer by regulating PTEN/Akt axis

**DOI:** 10.18632/aging.202919

**Published:** 2021-04-13

**Authors:** Zilian Cui, Hui Gao, Ning Yan, Yun Dai, Hanbo Wang, Muwen Wang, Jin Wang, Dong Zhang, Peng Sun, Taiguo Qi, Qiang Wang, Weiting Kang, Xunbo Jin

**Affiliations:** 1 Department of Urology, Shandong Provincial Hospital, Cheeloo College of Medicine, Shandong University, Jinan, Shandong 250021, China; 2 Department of Urology, Shandong Provincial Hospital Affiliated to Shandong First Medical University, Jinan, Shandong 250021, China; 3 Department of Urology, Liaocheng People′s Hospital, Liaocheng, Shandong 252000, China; 4 Department of Plastic Surgery, Jinan Central Hospital Affiliated to Shandong University, Jinan, Shandong 250013, China; 5 Department of Ultrasound, Shandong Provincial Hospital Affiliated to Shandong First Medical University, Jinan, Shandong 250021, China; 6 Department of Ultrasound, Shandong Provincial Hospital, Cheeloo College of Medicine, Shandong University, Jinan, Shandong 250021, China; 7 Department of Urology, The First Affiliated Hospital of Shandong First Medical University, Jinan, Shandong 250021, China; 8 Department of Urology, Shandong Provincial Qianfoshan Hospital, Cheeloo College of Medicine, Shandong University, Jinan, Shandong 250021, China; 9 Department of Human Resources, Shandong Provincial Hospital Affiliated to Shandong First Medical University, Jinan, Shandong 250021, China; 10 Department of Human Resources, Shandong Provincial Hospital, Cheeloo College of Medicine, Shandong University, Jinan, Shandong 250021, China

**Keywords:** long non-coding RNA, plncRNA-1, prostate cancer, PTEN

## Abstract

Long non-coding RNAs are key regulators of tumor development and progression, with the potential to be biomarkers of tumors. This study aimed to explore the role of PlncRNA-1 in the progression of prostate cancer (PCa). We found that PlncRNA-1 was up-regulated in 85.29% of PCa tissues and could predict the T stage of PCa patients to a certain extent. Results showed that inhibition of PlncRNA-1 expression potentially promoted cell apoptosis, suppressed the proliferation, migration, and invasion of cells, and triggered G2/M cycle arrest *in vitro* and *in vivo*. PlncRNA-1 was mainly localized in the nucleus and PlncRNA-1 expression and phosphatase and tensin homologue (PTEN) expression were negatively correlated. Mechanistically, knockdown of PlncRNA-1 increased expression levels of PTEN protein and phosphorylated PTEN protein, and decreased expression levels of Akt protein and phosphorylated Akt protein. Rescue experiments demonstrated that PTEN inhibitors abolished the changes in PTEN/Akt pathway caused by PlncRNA-1 interference. PlncRNA-1 can promote the occurrence and development of PCa via the PTEN/Akt pathway. PlncRNA-1 may, therefore, be a new candidate target for the treatment of PCa.

## INTRODUCTION

Prostate cancer is the second most common cancer and malignant tumor of the male genitourinary system. In 2020, the American Cancer Society reported an estimated 191,930 new cases of PCa, accounting for 21% of all male tumors and its incidence is the highest of all male tumors [1]. It results in an estimated 33,330 deaths in 2020, making it the second leading cause of cancer-related mortality in men [1]. Nowadays, radical prostatectomy and radiotherapy are the standard treatments for patients with localized PCa, while androgen suppression therapy is the main treatment for recurrent disease and advanced PCa [2]. Although androgen suppression therapy is initially effective, nearly all PCa patients eventually develop metastatic castration-resistant PCa [3]. The average overall survival of patients with metastatic castration-resistant PCa varies between 13 to 32 months, and the 5-year survival rate is less than 15% [4]. Despite the wide spread application of prostate-specific antigen in clinical screening, its low specificity leads to poor diagnosis and treatment [5]. Therefore, more sensitive biomarkers should be developed to improve early detection and diagnosis of PCa.

PTEN is a tumor suppressor gene that is frequently destroyed in a variety of cancers. PTEN, as the most important negative regulator of phosphatidylinositol 3 kinase (PI3K) signaling pathway, has been studied in various research fields due to its ability to regulate diverse physiological functions. Broadly, PTEN inhibits cell proliferation, cell survival, regulates genomic stability, cell migration, energy metabolism, cell structure, stem cell self-renewal, and tumor microenvironment [6, 7]. The expression and function of PTEN are altered in cancer [8]. In PCa, deletion of PTEN and alterations of PTEN-PI3K pathway activity have been reported in many advanced PCa [9, 10]. Loss of PTEN is strongly associated with poor prognosis of PCa [11]. Therefore, a comprehensive understanding of the pathological role of PTEN will undoubtedly lead to the rational design of new PCa therapies.

Long non-coding RNA (lncRNA) play diverse functions in Eukaryotes [12]. For instance, lncRNAs participate in physiological and pathological processes, including embryonic development, organ formation, and tumorigenesis [12–14]. Recent evidence shows that lncRNA regulate tumorigenesis and cancer progression by modulating the proliferation, metastasis, and invasion of cancer [14]. For instance, lncRNA lncAMPC promotes the metastasis and immunosuppression of PCa [15]. Also, lncRNA SNHG17 aggravates the proliferation, invasion, migration, and epithelial-mesenchymal transition of PCa [8]. In addition, lncRNA PCAT7 has been shown to promote bone metastasis of PCa [16]. LncRNA HORAS5 prolongs the survival of patients with castration-resistant PCa by activating androgen receptor transcription [17].

PlncRNA-1 (transcript variant 3 of CBR3-AS1) is highly expressed in PCa and modulates the proliferation and apoptosis of PCa [18]. Studies have shown that androgen receptor (AR) forms a regulatory feed-forward loop that drives the development of PCa. Notably, AR promotes the expression of PlncRNA-1, which in turn sponges microRNA that targets AR and protects AR from microRNA-mediated downregulation [19]. Studies have found that PlncRNA-1 is differentially expressed in a variety of diseases. PlncRNA-1 regulates cell proliferation, apoptosis, metastasis, epithelial-mesenchymal transition, autophagy and stem cell characteristics through multiple pathways [20–32]. Moreover, lncRNA has been shown to regulate cellular processes via different mechanisms, including chromatin remodeling, transcription and translation regulation, RNA stability, scaffolding, innate immunity, among other functions [33]. However, the mechanism of PlncRNA-1 in PCa is still not well understood. Whether PlncRNA-1 can promote the progression of PCa through PTEN remains elusive.

In this study, we characterized the biological functions of PlncRNA-1/PTEN in PCa. Expression of PlncRNA-1 in PCa tissues and its correlation with PTEN was evaluated using quantitative real-time polymerase chain reaction (qPCR). Further, we explored how PlncRNA-1 regulates the proliferation, migration, invasion, apoptosis and cell cycle. Finally, the effect of PlncRNA-1 on PTEN was examined using qPCR and western blot (WB) techniques. Our results reveal that PlncRNA-1 maybe a potential therapeutic target in PCa.

## MATERIALS AND METHODS

### Clinical tissue samples and patients’ data

In total, 34 pairs of PCa tissue and matched normal tissues samples were collected from the Department of Urology, Shandong Provincial Hospital, between May 2014 and June 2020. Among them, 18 cases received endocrine therapy before operation, while 16 cases did not. All 34 pairs of PCa tissues and matched normal tissues were used for qPCR analysis. All patients signed informed consent form, and the study was approved by the Ethics Committee of Shandong Provincial Hospital (Jinan, China).

### Culture of cell lines

Human PCa cell lines (DU145 and 22Rv1) were obtained from the Cell Bank of the Chinese Academy of Sciences (Shanghai, China). 22Rv1 and DU145 cells were cultured in RPMI-1640 (Gibco, CA, USA). All media were supplemented with 10% fetal bovine serum (Invitrogen, CA, USA) and 100 IU/ml penicillin (Sigma, MO, USA). Cell lines were maintained at 37°C and humidified atmosphere of 5% CO_2_.

### RNA isolation, reverse transcription and quantitative real-time PC

Total RNA was extracted from prepared tissues and cells using RNAiso Plus (Takara, Japan). The total RNA was reverse-transcribed into complementary DNA (cDNA) using the PrimeScript RT Reagent (Takara). PlncRNA-1 expression level was determined by qPCR reactions using the following primer sequences; forward, 5′- TGGCCAGGATCCTCGATAAGAC-3′, reverse 5′- CTTGTAGCCGCCAAGTTTCTGA-3′ using the SYBR Premix Ex Tap (Takara) on the LightCycler 480 II (Roche, Switzerland). Similarly, expression levels of PTEN was determined using primer sequences: forward, 5′-ACACCGCCAAATTTAATTGCAG-3′ and reverse, 5′-TGTCATCTTCACTTAGCCATTGGTC-3′. While, for Akt: forward 5′- AGCGACGTGGCTATTGTGAA-3′ and reverse 5′- CACGTTGGTCCACATCCTG-3′ primers were used. GAPDH mRNA served as the internal control. GAPDH primers used were: forward, 5′-ACACCGCCAAATTTAATTGCAG-3′ and reverse, 5′-ACACCGCCAAATTTAATTGCAG-3′. All experimental procedures were carried out in accordance to the manufacturer's protocol. Expression profiles obtained from qPCR results were analyzed using R program package ‘pcr’.

### Cell transfection

siRNA and negative control siRNA vectors targeting PlncRNA-1 were purchased from Sangon Biotech (Shanghai, China). The sequences were: Control siRNA: 5′-UUC UCC GAA CGU GUC ACG UTT-3′; PlncRNA-1 siRNA: 5′-GGC GGC UAC AAG GAA UUA ATT-3′. The vectors were transfected into DU145 and 22Rv1 cells using Lipofectamine 3000 (Invitrogen) according to the manufacturer's protocol. All transfection experiments were carried out within a period of 48 h. The efficiency of transfection was tested using qPCR analysis.

### Assessment of cell viability, migratory, invasion, apoptosis, and cell cycle

The Cell Counting Kit (CCK-8, Dojindo, Japan) assay was used to determine the proliferation of the prostate cancer cell lines. Cells were seeded in 96-well plates and they were then transfected siRNA. Cells were then cultured for 0, 24, 48, 72 and 96 h. At the end of the experiment, 10 μl CCK-8 solution was added into each well and the cells were incubated at 37°C in an incubator with 5% CO_2_ for 1 h. Spectrophotometric absorbance values of each sample were recorded at 450 nm using Spectrophotometer Multiskan Go (Thermo Fisher Scientific, Finland). The wound healing assay was used to assess the migration of PCa cells. Briefly, transfected prostate cancer cells were grown to 90% confluence in a six-well plate. A wound was created with a 200 μl sterile pipette tip. The cells were incubated at 37°C with 5% CO_2_ and imaged at 0 h, 24 h and 48 h. Transwell assays were performed to determine the migration and invasion of prostate cancer cell lines. After transfection, 3 × 10^4^ cells in serum-free medium were seeded on uncoated upper chambers (Costar, NY, USA) to measure cell migration ability. For the invasion assay, 5 × 10^4^ cells in serum-free medium were seeding on Matrigel-coated (BD Bioscience, CA, USA) upper chambers (Costar, NY, USA). A culture medium containing 10% FBS was added into the lower wells and incubated for further 24 h. Cells in three random fields were counted for the determination of cell migration and invasion. Flow cytometric analysis was performed to determine the cell apoptosis and cell cycle in the prostate cancer cell lines. After transfection with the siRNA or negative control in 6-well plates, the cells were cultured for 48 h. Next, FITC Annexin V Apoptosis Detection Kit (BD Biosciences) was used to analyze cell apoptosis according to the manufacturer's instructions, and DNA Content Quantitation Assay (Cell Cycle) (Solarbio, Beijing, China) was used to analyze cell cycle.

### Nude mouse xenograft model

All animals experiments were approved by the Institutional Animal Care and Use Committee of Shandong Provincial Hospital. 1 × 10^7^ cells/0.1 ml DU145 single-cell suspension was injected subcutaneously into 4-week-old male BALB/c nude mice (Vital River, Beijing, China) for tumor xenotransplantation experiments. The length (L), width (W), and estimate of the height (H) of the subcutaneous xenograft tumor were measured every 3 days. The volume of each tumor was calculated according to the formula: V = π/6*(L*W*H), from which a tumor growth curve was drawn. After 4 weeks, nude mice were humanely sacrificed, and tumors were excised, weighed, and imaged.

### Western blotting analysis

Protein samples were extracted from cells using RIPA Lysis Buffer (Beyotime, Shanghai, China). The proteins were separated on 10% SDS-PAGE and transferred to a PVDF membrane. The membrane was blocked with 5% skimmed milk solution for 1 hour. The membrane was treated with the following primary antibodies: anti-PTEN (Cat. #9188, Cell Signaling Technology, Danvers, MA), anti-p-PTEN (Cat. #9549, Cell Signaling Technology), anti-Akt (ab8805, Abcam, MA, USA), anti-p-Akt (ab38449, Abcam), anti-E-cadherin (Cat. #3195, Cell Signaling Technology), anti-E-cadherin (Cat. #13116, Cell Signaling Technology) and anti-vimentin (ab92547, Abcam) and corresponding secondary antibodies conjugated with horseradish peroxidase. Protein bands were visualized using an enhanced chemiluminescence system (Millipore, Bedford, MA) and analyzed with Amersham image 800 (GE). The GAPDH protein (Cat. #5174, Cell Signaling Technology) served as the loading control.

### RNA fluorescent *in situ* hybridization (FISH)

PlncRNA-1 FISH probes were designed and synthesized by the RiboBio Company (Guangzhou, China). The DU145 cells were collected after transfection for 48 h and mounted on glass coverslips. RNA FISH was performed using a fluorescent *in situ* hybridization kit (RiboBio) following the manufacturer’s instructions. Finally, a fluorescence scanning microscope (Leica, Germany) was used to measure fluorescence of cells.

### Immunohistochemistry (IHC)

Tissue samples were paraffinized and analyzed using SP Link Detection kit (Rabbit Biotin-streptavidin HRP Detection Systems) as per the manufacturer’s instructions (ZSGB-Bio, Beijing, China). The sections were visualized under a fluorescent microscope (Vienna, Austria). The image pro plus software was calculated the integrated optical density (IOD) value of immunohistochemistry.

### Statistical analysis

All statistical analyses and data visualization were performed using R 3.6.1. The R package ‘edgeR’ was used for differential analysis while R package‘ggplot’ was used to visualize histograms, box plots and line plots. Student’s *t*-test, ANOVA, Spearman’s rank correlation test and χ^2^ test were used for statistical analysis. Data were presented as the mean ± SD of three independent experiments. A *p* < 0.05 was considered statistically significant.

## RESULTS

### Differential expression of PlncRNA-1 in prostate cancer

A total of 34 pairs of PCa and matched normal tissues were collected from the Department of Urology, Shandong Provincial Hospital from March 2014 to June 2020. As shown in Table 1, 18 patients received endocrine therapy before operation while 16 patients did not. The average age of patients was 68.147 ± 6.977 years old with a preoperative prostate-Specific Antigen (PSA) level of 25.335 ± 29.737. From the results, 20 patients recorded a Gleason score of 6–7, while 14 patients had Gleason score of 8–9. In 19 patients, tumors were localized in the prostate, whereas in 15 patients, tumors extended beyond the prostate. Additionally, 6 cases of PCa patients with lymph node metastasis and 28 cases without lymph node metastasis were found.

**Table 1:** The correlation between the expression levels of PlncRNA-1 and clinicopathological features of PCa patients.

**Characteristics**		**Total**	**PlncRNA-1**		***p* value**
			**Low**	**High**	
Age (year)		68.147 ± 6.977	67.647 ± 7.088	68.647 ± 7.044	0.683
Preoperative treatment	None	16	7	9	0.731
	Endocrine therapy	18	10	8	
Preoperative tPSA		25.335 ± 29.737	18.741 ± 28.888	32.399 ± 30.033	0.223
Gleason	6–7	20	10	10	1.000
	8–9	14	7	7	
Gleason primary grade	3	11	7	4	0.721
	4	21	9	12	
	5	2	1	1	
Gleason secondary grade	3	12	5	7	0.885
	4	18	10	8	
	5	4	2	2	
T Stage	T2	19	13	6	0.038
	T3-T4	15	4	11	
N stage	N0	28	14	14	1.000
	N1	6	3	3	

**Figure 1:**
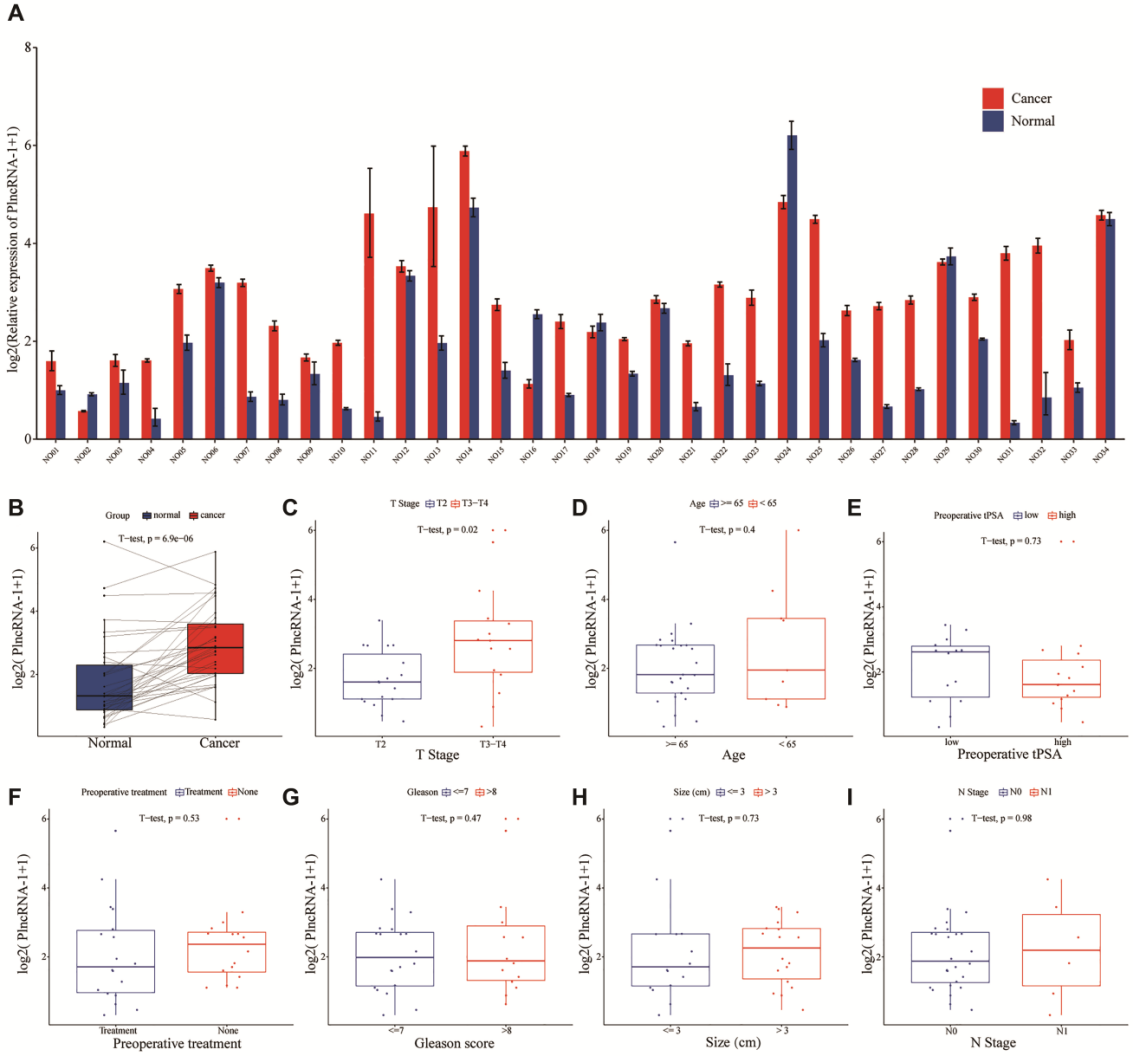
**The level of PlncRNA-1 expression in PCa tissues.** (**A**–**B**) The expression level of PlncRNA-1 in 34 pairs of PCa and normal matched tissues. (**C**–**I**) The correlation between the expression level of PlncRNA-1 and T stage, age, preoperative tPSA, preoperative treatment, Gleason score, tumor size, and N stage.

Compared with normal tissues, PlncRNA-1 mRNA level was significantly elevated in 85.29% (29/34) PCa patients (*p* < 6.9e-06) (Figure 1A–1B). Subgroup analysis showed that the expression level of PlncRNA-1 in PCa patients with T3-T4 stage was significantly higher than in those with T2 stage (*p* < 0.02) (Figure 1C). However, the expression level of PlncRNA-1 was not significantly related with age, total preoperative PSA level, preoperative treatment, Gleason score, tumor size, and lymph node metastasis of PCa patients (Figure 1D–1I). PCa patients were divided into high and low expression levels of PlncRNA-1 based on the median value of 2.696. As noted from Table 1, the proportion of patients with T3-T4 PCa was 64.71% (11/16) in the high PlncRNA-1 expression group, whereas the proportion of patients with T3-T4 PCa was 23.53% (4/17) in the low PlncRNA-1 expression group, and the difference was significant (*p* < 0.038). In other words, if a patient's expression level of PlncRNA-1 exceeded 2.696, the probability of the patient being diagnosed with T3-T4 staging was 64.71%. Conversely, if a patient's expression level of PlncRNA-1 was less than 2.696, the probability of the patient being diagnosed with T3-T4 staging was 23.53%. PlncRNA-1 showed the potential to predict the T stage of PCa patients to a certain extent. In the high and low PlncRNA-1 expression groups, age, preoperative treatment, preoperative PSA level, Gleason score, and lymph node metastasis were not correlated with PlncRNA-1 expression. Hence, the expression level of PlncRNA-1 may be utilized as a predictor of the clinical stage of PCa.

### PlncRNA-1 promotes the proliferation, migration and invasion of PCa cells *in vitro*

**Figure 2:**
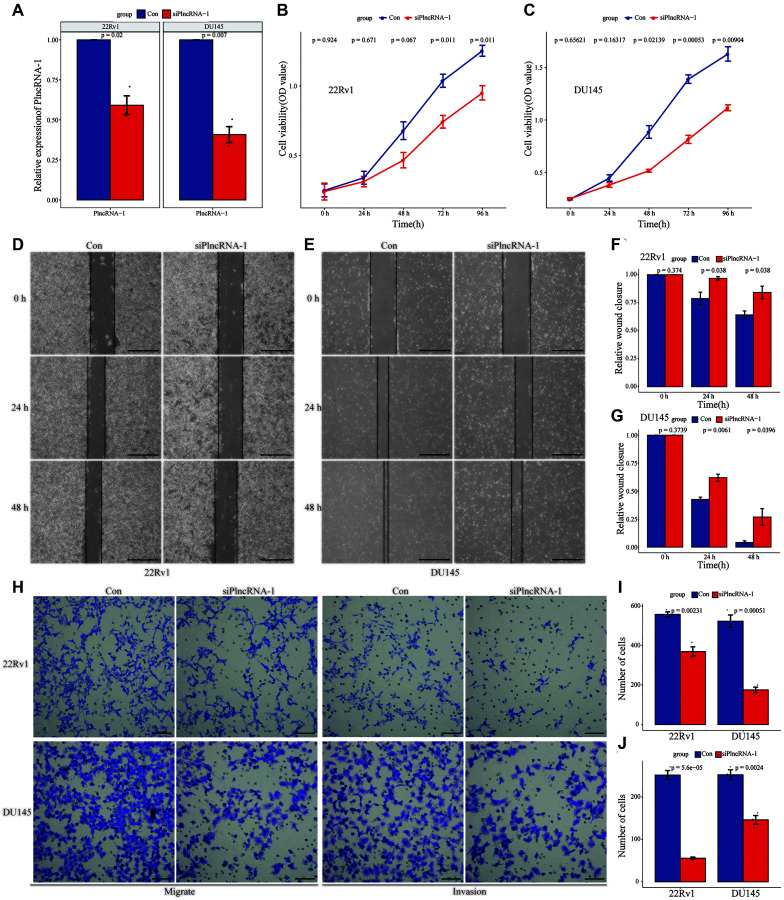
**Effect of PlncRNA-1 knockdown on the proliferation, migration, and invasion of PCa cells.** (**A**) qPCR analysis for the transfection efficiency of PlncRNA-1-siRNA in 22Rv1 and DU145 PCa cells. (**B**–**C**) CCK-8 assay for the proliferation of 22Rv1 and DU145 PCa cells after PlncRNA-1 silencing. (**D**–**G**) Wound healing assays for the migration of 22Rv1 and DU145 PCa cells after PlncRNA-1 silencing (Scale bar, 200 μm). (**H**) Transwell migration and invasion assays for the migration and invasion of 22Rv1 and DU145 PCa cells after PlncRNA-1 silencing (Scale bar, 100 μm). (**I**–**J**) Quantitative analysis transwell migration (**I**) and invasion (**J**) assays of 22Rv1 and DU145 PCa cells after PlncRNA-1 silencing.

As an oncogene, PlncRNA-1 is highly expressed in PCa tissues. Therefore, siRNA was synthesized to interfere with the expression of PlncRNA-1 in PCa DU145 cells and 22Rv1 cell lines. Compared with the control group, the expression level of PlncRNA-1 in the siPlncRNA-1 group was significantly lower indicating that siRNA effectively reduced the expression of PlncRNA-1 in PCa DU145 cells and 22Rv1 cell lines (Figure 2A). Moreover, results of the CCK-8 showed that PlncRNA-1 significantly reduced the proliferation ability of DU145 cells and 22Rv1 cells *in vitro* (Figure 2B–2C). The wound healing assays demonstrated that the migration ability of DU145 cells and 22Rv1 cells began to decrease at 24 hours after silencing PlncRNA-1 expression (Figure 2D–2G). After 48 hours of transfection, the migration ability of PCa DU145 cells and 22Rv1 cells was significantly reduced. The transwell experiment revealed that the migration and invasion of PCa DU145 cells and 22Rv1 cells were inhibited following PlncRNA-1 silencing (Figure 2H–2J). These findings indicate that inhibition of PlncRNA-1 *in vitro* can significantly inhibit the proliferation, migration and invasion of PCa cells.

### PlncRNA-1 regulates cell apoptosis and cell cycle *in vitro*

**Figure 3:**
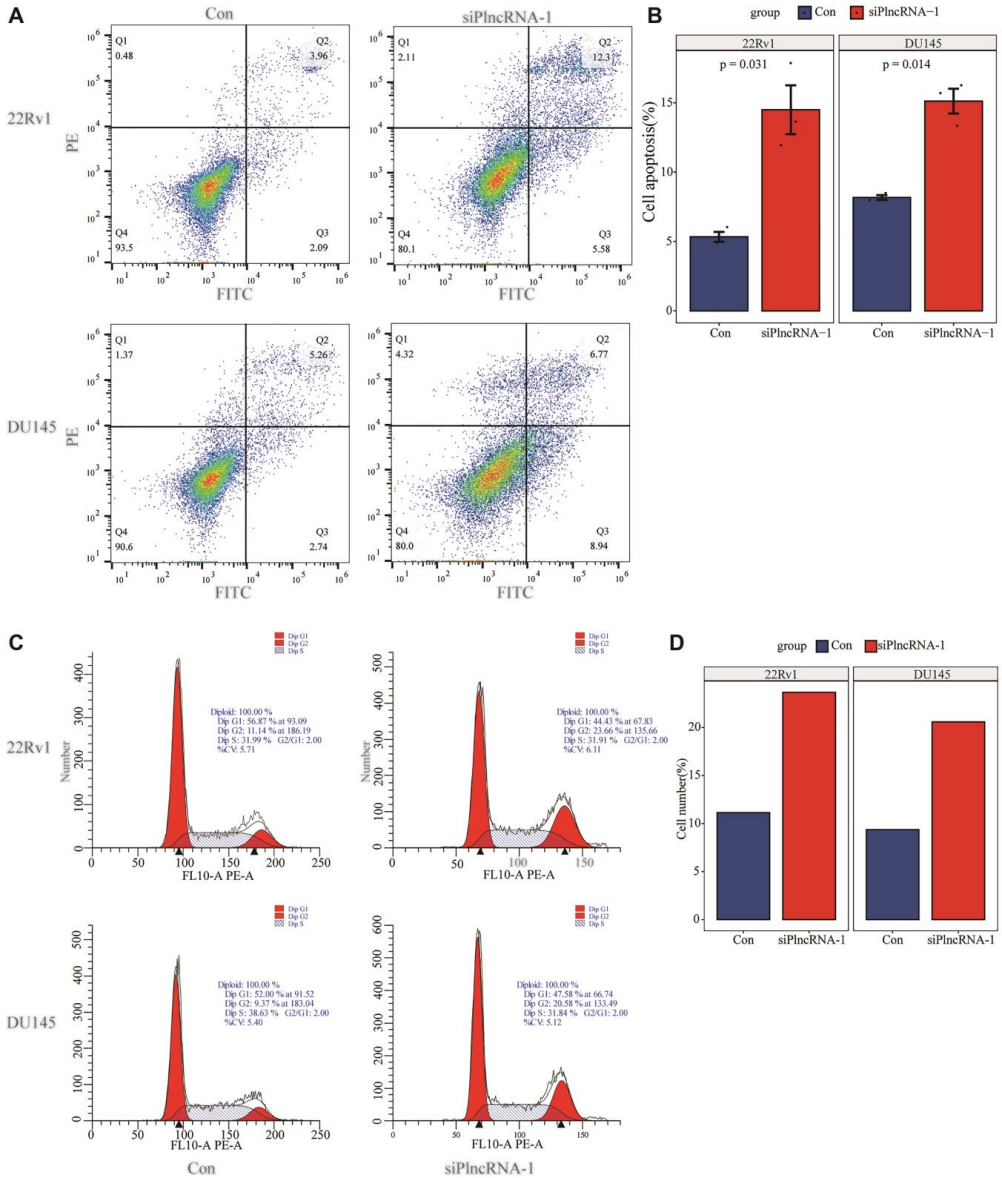
Flow cytometry detects the changes in apoptosis (**A**–**B**) and cell cycle (**C**–**D**) in 22Rv1 and DU145 PCa cells after interference with PlncRNA-1.

Flow cytometry was used to detect the effect of PlncRNA-1 on cell apoptosis. The number of apoptotic 22Rv1 cells increased from 6.05% to 17.88% following PlncRNA-1 silencing compared with the control group and the difference was statistically significant (*P* < 0.031) (Figure 3A and 3B). Similarly, the number of apoptotic DU145 cells increased from 8.00% to 15.71% (*p* < 0.014) after silencing PlncRNA-1 expression (Figure 3A and 3B). This indicated that silencing PlncRNA-1 expression promoted apoptosis of PCa cells. Further analysis showed that PlncRNA-1 blocked the cell cycle of 22Rv1 cells at G2/M cycle. Notably, the number of cells in the G2/M cycle increased from 11.14% to 23.66% following PlncRNA-1 silencing (Figure 3C and 3D). Similarly, the number of DU145 cells in the G2/M cycle increased from 9.37% to 20.58% following PlncRNA-1 silencing (Figure 3C and 3D). These results show that silencing PlncRNA-1 expression can cause G2/M cycle arrest in PCa cells.

### Silencing PlncRNA-1 expression inhibits the tumorigenicity of PCa cells *in vivo*

**Figure 4:**
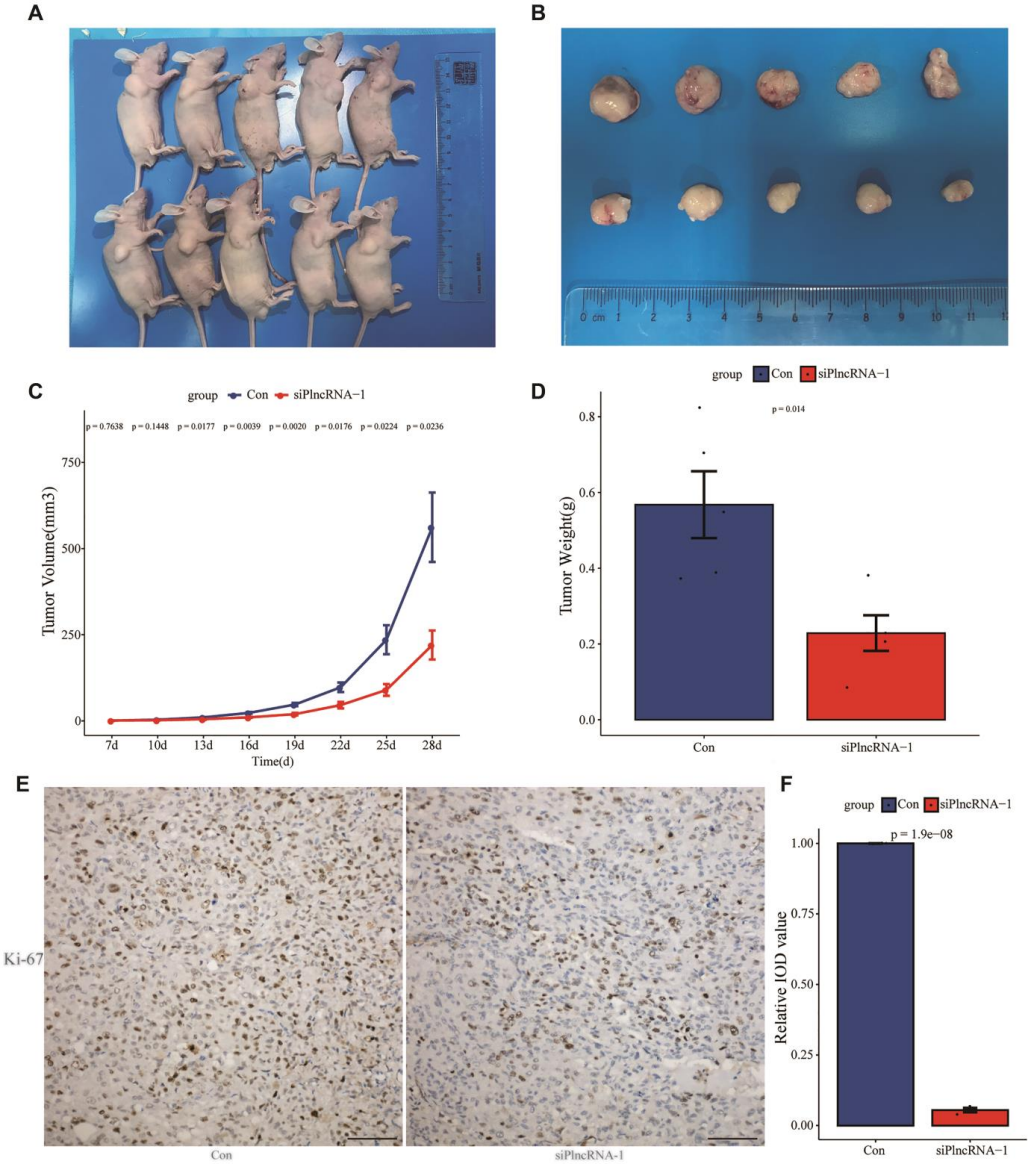
**Xenograft tumor formation of DU145 cells in PlncRNA-1-silenced nude mice.** (**A**) Photograph of nude mice with Xenografted tumors on day 28. (**B**) Pictorials of subcutaneous tumors on day 28. (**C**) Growth curves for xenografted tumors. (**D**) The tumor weights in different groups. (**E**) Immunohistochemistry assay for the expression of Ki-67 in the tumor xenografts (Scale bar, 100 μm). (**F**) Quantitative analysis immunohistochemistry assay for the expression of Ki-67 in the tumor xenografts.

DU145 cells in which PlncRNA-1 was silenced were subcutaneously injected into nude mice. This resulted in inhibition of tumor growth after 4 weeks compared with the control group (Figure 4A–4B). Silencing PlncRNA-1 expression decreased the volume (Figure 4C) and weight (Figure 4D) of the implanted tumor. IHC analysis of the implanted tumor showed that silencing PlncRNA-1 expression inhibited Ki-67 expression (Figure 4E–4F). These results proved that inhibiting PlncRNA-1 expression *in vivo* significantly inhibited the tumorigenicity of PCa cells.

### Correlation between PlncRNA-1 and PTEN expression

**Figure 5:**
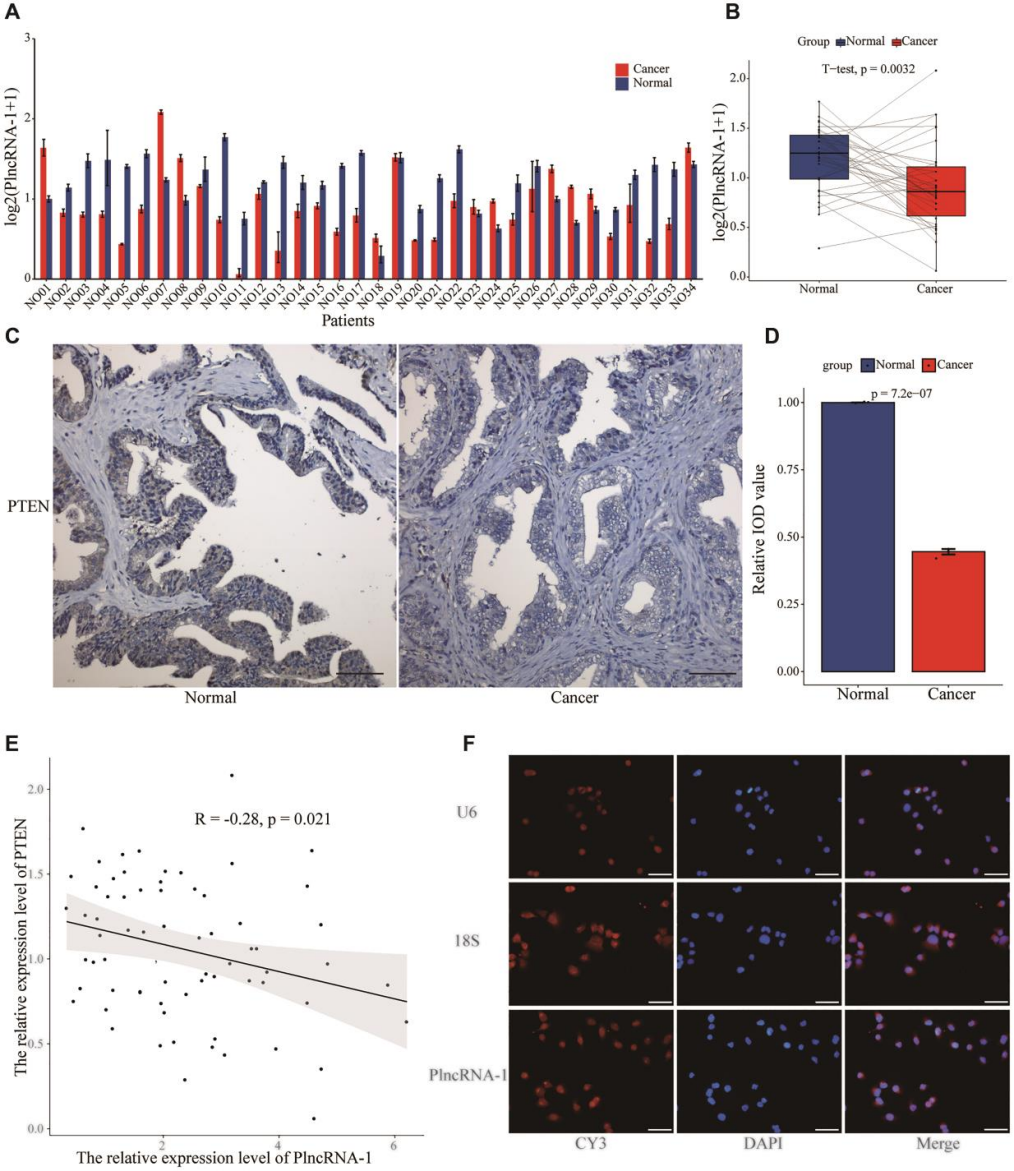
**The relationship between PTEN and PlncRNA-1**. (**A**–**B**) The expression level of PTEN in 34 pairs of PCa and normal matched tissues. (**C**) Immunohistochemistry assay for the expression of PTEN in the PCa and normal matched tissues (Scale bar, 100 μm). (**D**) Quantitative analysis immunohistochemistry assay for the expression of PTEN in the PCa and normal matched tissues. (**E**) The correlations between level of PlncRNA-1 expression and PTEN in PCa tissues. (**F**) RNA-FISH images for subcellular localization of PlncRNA-1 in PCa cells (Scale bar, 50 μm).

*In vivo* and *in vitro* experiments showed that PlncRNA-1 regulated the proliferation, migration, invasion, apoptosis and cell cycle of PCa. However, it was not clear whether PlncRNA-1 could down-regulate PTEN. Thus, we assessed the relationship between PlncRNA-1 and PTEN in 34 pairs of PCa tissues. The mRNA expression level of PTEN in 67.65% (23/34) of PCa tissues was significantly lower than that of normal tissue (*p* < 0.0032), (Figure 5A–5B). Similarly, the expression level of PTEN protein in PCa tissue was lower than in normal tissue as revealed by IHC (Figure 5C–5D). A negative relationship was found between PlncRNA-1 and PTEN expression (R = –0.28, *p* < 0.021) in PCa tissues (Figure 5E)

RNA Fish was used to locate the position of PlncRNA-1 in the cell. We choose U6 and 18S as internal reference genes. U6, exhibiting a red Cy3 fluorescence was distributed in the nucleus, and 18S with a red Cy3 fluorescence was distributed in the cytoplasm. We found that PlncRNA-1 was mainly distributed in the nucleus and partly distributed in the cytoplasm (Figure 5F). PTEN is normally distributed in the nucleus and cytoplasm, almost similar to PlncRNA-1. Based on the similar subcellular localization and negative relationship between PlncRNA-1 and PTEN, we speculate that they may be mutually regulated.

### PlncRNA-1 regulates PTEN/Akt pathway in prostate cancer cells

**Figure 6:**
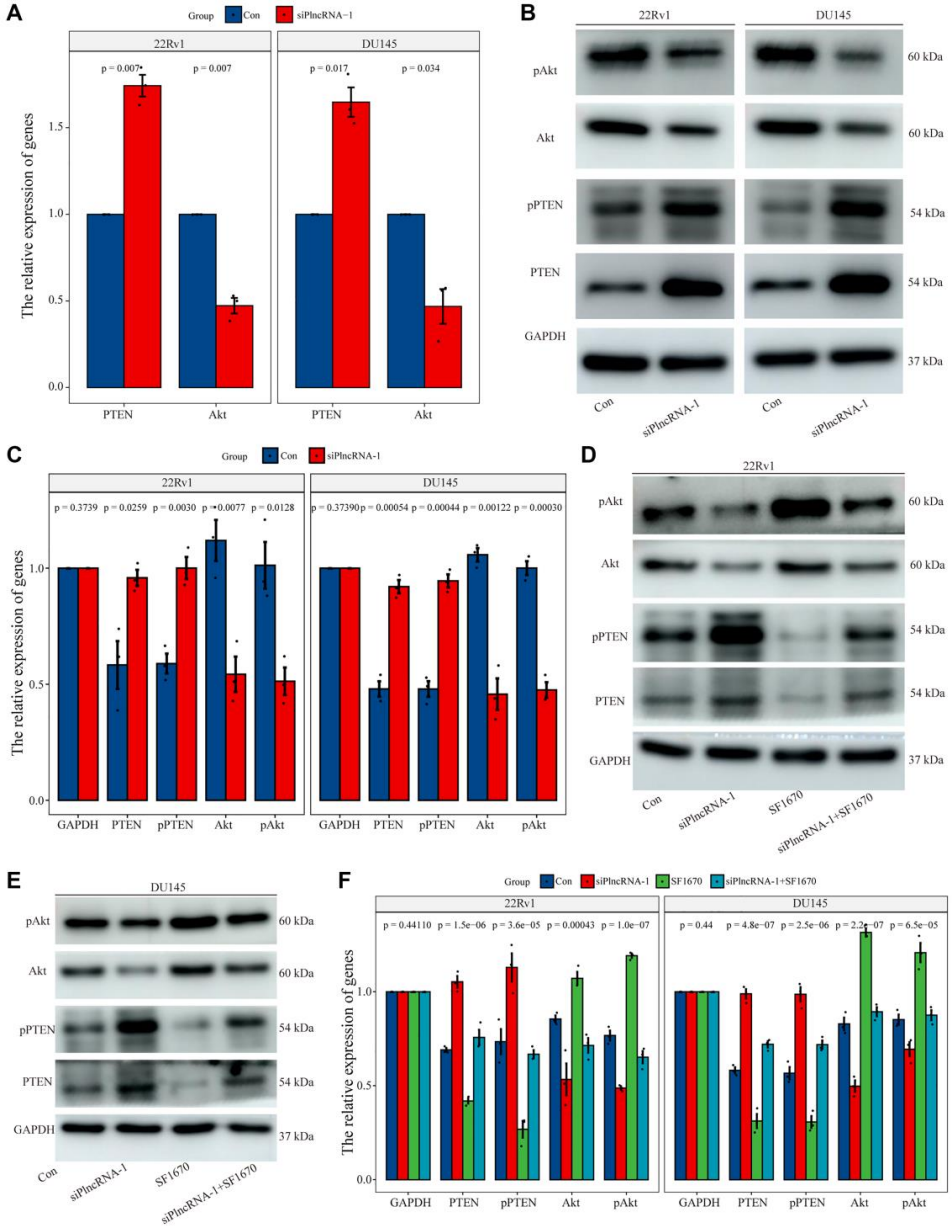
**Regulation of PTEN expression by PlncRNA-1.** (**A**) qPCR analysis for the expression level of PTEN and Akt in PlncRNA-1 silent PCa cells. (**B**) Western blot for the expression level of PTEN and Akt in siPlncRNA-1 group. (**C**) The histogram shows the statistical analysis of Western blot in Figure 6B. (**D**–**E**) The relative expression levels of PTEN and Akt protein were detected in difference group by Western blot. (**F**) The histogram shows the statistical analysis of Western blot in Figure 6D–6E.

To determine the regulatory mechanism between PlncRNA-1 and PTEN, we silenced PlncRNA-1 expression in DU145 and 22Rv1 cells to measure the expression levels of PTEN and Akt with qPCR and WB. Notably, expression level of PTEN mRNA increased, whereas the expression level of Akt mRNA decreased after PlncRNA-1 silencing (Figure 6A). The protein level of PTEN and phosphorylated PTEN were increased after PlncRNA-1 silencing in the PCa DU145 and 22Rv1 cells (Figure 6B–6C). However, the expression levels of Akt protein and phosphorylated Akt protein were decreased after PlncRNA-1 silencing in PCa DU145 and 22Rv1 cells (Figure 6B–6C).

To further explore whether the function of PlncRNA-1 was mediated via the PTEN/Akt axis, we conducted a rescue experiment in the PCa DU145 and 22Rv1 cells. The experiment was performed in four groups: control group, siPlncRNA-1 group, PTEN inhibitor (SF1670) group and the siPlncRNA-1+SF1670 group. In order to verify the efficiency of SF1640 in inhibiting PTEN/ Akt pathway, compared with the control group, the expression levels of PTEN protein and phosphorylated PTEN protein decreased, whereas the expression levels of phosphorylated Akt protein increased in SF1670 group (Figure 6D–6F), which providing evidence that PTEN inhibitors could effectively inhibit PTEN/ Akt pathway. Compared with the control group, silencing PlncRNA-1 expression with PCa DU145 and 22Rv1 cells increased PTEN protein and phosphorylated PTEN protein levels but decreased the expression levels of Akt protein and phosphorylated Akt protein in siPlncRNA-1 group (Figure 6D–6F). Compared with siPlncRNA-1 group, treatment with PTEN inhibitor in siPlncRNA-1 group significantly inhibited the upregulation of PTEN and phosphorylated PTEN induced by PlncRNA-1 interference and promoted the downregulation of Akt and phosphorylated Akt induced by PlncRNA-1 interference (Figure 6D–6F). Collectively, these results showed that PlncRNA-1 regulated PTEN/Akt axis in PCa cell lines.

## DISCUSSION

Prostate cancer is a common urological tumor and a leading cause of cancer-related deaths in men [34]. Most PCa are androgen-dependent, and hence androgen deprivation therapy is considered the standard first-line treatment for advanced PCa. This is achieved through surgical castration, medical castration, anti-androgen and androgen biosynthesis inhibitors. These therapies effectively relieve symptoms, reduce tumor burden, and prolong patient survival. However, it is unfortunate that hormone deprivation therapy rarely cures cancer itself because most PCa cases recur, leading to deadly castration-resistant PCa [35]. This calls for further studies to determine the mechanism of PCa occurrence and development.

Previous studies have shown that several lncRNAs are dysregulated in tumors, and these affects tumorigenesis and tumor progression [36, 37]. The level of lncRNAs can reflect the stage of tumor development in PCa patients [38]. Our study found that the expression level of PlncRNA-1 was significantly higher in 85.29% PCa tissues. Analysis of clinical information of PCa patients showed that PlncRNA-1 was related to the T stage of PCa patients. Consequently, PlncRNA-1 was found to be highly expressed in patients with T3-T4 stage as compared to those with T2 stage. In addition, the proportion of patients with T3-T4 PCa was 64.71% in the high PlncRNA-1 expression group, whereas the proportion of patients with T3-T4 PCa was 23.53% in the low PlncRNA-1 expression group. In other words, if a patient's expression level of PlncRNA-1 exceeded 2.696, the probability of the patient being diagnosed with T3-T4 staging was 64.71%. Conversely, if a patient's expression level of PlncRNA-1 was less than 2.696, the probability of the patient being diagnosed with T3-T4 staging was 23.53%. Therefore, PlncRNA-1 can be used to predict the T staging of PCa. This should be verified in larger sample size.

Several studies have shown that lncRNA-1 can regulate various biological processes in PCa. For instance, lncRNA DSCAM-AS1 and LINC00675 promote the progression of castration-resistant PCa [39], lncRNA SNHG17 regulates the proliferation, invasion, migration, epithelial-mesenchymal transition and apoptosis of PCa cells [8], and lncRNA PCAT7 promotes bone metastasis of PCa [16]. In the present study, we found that *in vitro* silencing of PlncRNA-1 expression significantly inhibited the proliferation, migration and invasion of PCa cells, promoted cell apoptosis, and caused G2/M cycle arrest. *In vivo* experiments confirmed that PlncRNA-1 expression significantly decreased the weight and volume of the implanted tumor as well as decreased expression of Ki-67, indicating that *in vivo* silencing of PlncRNA-1 significantly reduced proliferation ability of PCa cells.

*In vivo*, lncRNA regulates a plethora of different cellular processes: chromatin remodeling, regulation of transcription and translation, RNA stability, scaffolding, and innate immunity among other processes [33]. A single lncRNA can act via multiple signaling pathways. For example, lncRNA HOTAIR acts through miR-204-5p/HMGB1 axis [40], miR-519a-3p/RRM1 axis [41], miR-601/ZEB1 axis [42], JAK2/STAT3 [43], hexokinase-2 [44], PI3K/Akt/mTOR [45] among other pathways. PlncRNA-1, is a newly identified lncRNA located on human chromosome 21 [18]. PlncRNA-1 can promote the occurrence and development of PCa through AR, human epidermal growth factor receptor 2 (Her-2) and Transforming growth factor beta 1 (TGF-β1) pathways [19, 24, 32] but whether PlncRNA-1 regulates other pathways remains unclear.

PTEN is a classic tumor suppressor that regulates PI3K/Akt signaling cascade. The phosphatidylinositol 4,5-bisphosphate (PIP2) is converted to phosphatidylinositol 3,4,5-trisphosphate (PIP3) by PI3K. Thereafter, PIP3 recruits phosphatidylinositol-dependent kinase 1 (PDK1) and Akt to the plasma membrane, where Akt is phosphorylated on Thr308 by PDK1 and on Ser473 by the mammalian target of rapamycin complex 2 (mTORC2) [46, 47]. PTEN reverses the effect of PI3K by phosphorylating PIP3 to PIP2, thereby blocking all downstream functions regulated by the Akt/mTOR axis, such as cell death, transcription, translation, stimulating angiogenesis and stem cell self-renewal [6, 48–53]. In PCa, genetic changes (e.g., activation mutation or deletion of PIK3CA, Akt1 and PTEN, epigenetic and post-translational modifications) leads to dysregulation of PI3K pathway and hence regulates cancer progression, including PCa [54]. Drugs targeting the PI3K pathway are limited by acute reactivation or drug resistance resulting from crosstalk between PI3K pathway and AR or other signaling networks [34]. PlncRNA-1 and AR form a feedforward regulatory loop that promotes the progression of PCa [19]. PlncRNA-1 appears to be a promising therapeutic target for PCa.

Furthermore, this study shows that the expression level of PTEN was lower in 67.65% PCa tissues than in normal tissues. Correlation analysis revealed an inverse relationship between the expression levels of PlncRNA-1 and PTEN. Results of RNA FISH assay showed that PlncRNA-1 was mainly localized in the nucleus, and a partly in the cytoplasm. Hence, the distribution of PTEN and PlncRNA-1 was nearly similar. Based on the above results, we postulate that PlncRNA-1 and PTEN may interact directly or indirectly. Subsequently, we silenced PlncRNA-1 expression in PCa cells which increased expression of PTEN protein and phosphorylated PTEN protein, and decreased expression levels of Akt protein and phosphorylated Akt protein. It was further observed that treatment with PTEN inhibitors alleviated the changes in the PTEN/Akt pathway caused by PlncRNA-1 silencing.

## CONCLUSIONS

These findings demonstrate that PlncRNA-1 is up-regulated in PCa tissues and it can predict T stage of PCa patients. In addition, silencing PlncRNA-1 inhibits the proliferation, migration and invasion of PCa cells, promotes apoptosis, and causes G2/M cycle arrest *in vitro* and *in vivo.* Mechanistic studies showed that the effects of PlncRNA-1 in prostate cancer were mediated by the PTEN/Akt axis. Therefore, this study reveals that PlncRNA-1 has a significant predictive, diagnostic or therapeutic value in PCa.
